# Sensory neuron–specific block of multifaceted sodium channels mitigates neuropathic pain behaviors of osteoarthritis

**DOI:** 10.1097/PR9.0000000000001288

**Published:** 2025-05-27

**Authors:** Seung Min Shin, Brandon Itson-Zoske, Hao Xu, Hongfei Xiang, Fan Fan, Quinn H. Hogan, Hongwei Yu

**Affiliations:** a Department of Anesthesiology, Medical College of Wisconsin, Milwaukee, WI, USA; b Department of Orthopedic Surgery, Affiliated Hospital of Qingdao University, Qingdao, China; c Department of Physiology, Medical College of Georgia, Augusta University, Augusta, GA, USA

**Keywords:** Sodium channels, Peripheral nervous system, Dorsal root ganglia, Sensory neurons, Adeno-associated virus, Osteoarthritis, Pain

## Abstract

**Objective:**

Multiple voltage-gated sodium channels (Na_V_s) in the peripheral sensory neurons (PSNs) regulate action potentials and their dysfunction contributes to the pain pathogenesis of osteoarthritis (OA). A combined block of multiple Na_V_ subtypes selectively in the PSNs may, therefore, represent an effective analgesic approach in OA painful neuropathy.

**Methods:**

To test this hypothesis, we generated recombinant adeno-associated virus (AAV) encoding a potent Na_V_ inhibitory peptide aptamer, termed Na_V_iPA1, that has a multipronged feature of inhibiting tetrodotoxin-sensitive Na_V_1.7, 1.6, 1.1, and 1.3, characterized in our recent report. Adeno-associated virus-encoded Na_V_iPA1 was delivered into the ipsilateral lumbar 4/5 dorsal root ganglia of rats 2 weeks after induction of knee monoiodoacetate-OA (MIA-OA) and evoked and spontaneous sensory behaviors were followed in 6 weeks.

**Results:**

Expression of Na_V_iPA1 selective in the PSNs produced significant and comparable mitigations of evoked and spontaneous pain behavior and reversal of weight-bearing asymmetry in both male and female MIA-OA rats. Whole-cell current-clamp recordings showed that AAV-mediated Na_V_iPA1 expression normalized action potential firing of the PSNs from MIA animals, suggesting that Na_V_iPA1 attenuated pain behavior by, at least in part, reversing neuronal hyperexcitability.

**Conclusion:**

Together, these results support that (1) Na_V_s in peripheral sensory pathways contribute to MIA-OA pain pathogenesis and (2) Na_V_iPA1 is a promising analgesic lead that, combined with AAV-targeted delivery to pathological sensory ganglia, may be a viable peripherally selective PSN-targeting strategy in mitigating chronic MIA-OA pain behaviors.

## Abstract 2

Supplemental Digital Content is Available in the Text.

Adeno-associated virus-mediated expression of multiple pronociceptive Nav peptide inhibitory peptide aptamer Na_V_iPA1 in peripheral sensory neurons attenuates the stimulated and spontaneous pain in osteoarthritis.

**Commentary on:**

Perez-Miller S, Khanna R. Beyond single targets: leveraging degeneracy in sodium channels for osteoarthritis analgesia. PAIN Rep 2025. DOI: 10.1097/PR9.0000000000001289.

## 1. Introduction

Injection of monoiodoacetate (MIA) into the articular cavity of the knee is an established preclinical osteoarthritis (OA) pain model.^34^ Pathogenesis of pain in MIA-OA constitutes a multifaceted and time-dependent interrelated pathological process, involving joint damage, sensory neuronal pronociceptive ion channel sensitization, structural reorganization of joint afferents, low-grade inflammation, and nerve damage-induced painful neuroplasticity in the sensory pathways, all contributing to the MIA-OA pain.^15,16,26^

Monoiodoacetate-osteoarthritis exhibits primary sensory neuron (PSN) and spinal cord neuron sensitization that resembles neuropathic pain conditions, suggesting a mechanistic overlap in these 2 pathologies.^63^ Many studies focus on changes in joint nociceptors and summate that OA pain is generated and maintained through continuous distinct peripheral nociceptive input from the affected joint to their innervating PSNs.^63^ These mechanisms include sensitization of joint afferents by local inflammation and release of inflammatory mediators, bone marrow lesions and microfractures, subchondral bone remodeling, and increased intraosseous pressure.^61^ However, clinical observation suggests a widespread nociceptive sensitization that includes joint afferents and PSNs innervating tissues outside the arthritic joint. Most patients with OA experience pain from the arthritic joint and referred pain from the areas remote from the OA joint, and pain severity in patients with OA is often not directly correlated with the degree of joint pathology.^25^ Clinical findings also support that neuropathic pain is prevalent in patients with end-stage hip and knee OA,^14,49,71^ and approximately 20% of patients experience chronic pain after total knee arthroplasty.^68^ Recently, an increasing focus has been on the combined involvement of both arthritic and neuronal pathology, indicating that PSN plasticity and morbidity contributes a critical component to persistent OA neuropathic pain.^15,40,62^

Monoiodoacetate-osteoarthritis produces nerve-injury responses with PSN damage as time progresses.^44^ This leads to the dysfunction of peripheral sensory pathways beyond exclusive sensitization of joint afferents and associated innervating PSNs,^67^ and adjacent nonjoint innervating PSNs including C-, Aδ-, and Aβ-neurons are also extensively affected, which could induce sensitization of affected joint, dermatomes, and increased nociceptive input to the dorsal horn of spinal cord.^28,67^ These comorbidities of joint- and nonjoint-innervating PSN sensitization are recognized as one of the essential pathologies for OA neuropathic pain-like behaviors.^40,62^ Importantly, joint-MIA–induced inflammatory and neuronal pathology in the PSNs can maintain pain independent from the damage of the joint in the late pathological stage,^52,70^ which may significantly influence the painful neuropathic course.

Aberrant expression of Na_V_s with heightened activity in PSNs contributes to the hyperexcitability of PSNs in OA.^16,51^ Here, we test whether a combined block of several Na_V_s selective in PSNs will mitigate OA-pain behaviors. We generated adeno-associated virus (AAV)-encoded Na_V_ inhibitory peptide aptamer (Na_V_iPA1) that shows multipronged inhibition of tetrodotoxin (TTXs)-Na_V_ in our prior study.^10,57^ Adeno-associated virus-mediated, PSNs-specific expression of Na_V_iPA1 in vivo produced significant attenuation of the pain behaviors in both male and female MIA-OA rats. Action potential firing of PSNs in MIA rats was normalized after treatment, suggesting that Na_V_iPA1 attenuated pain by reversal of injury-induced neuronal hypersensitivity.

## 2. Materials and methods

### 2.1. Animals and study approval

Experiments were performed in adult male and female Sprague Dawley rats (5–6 weeks old; 125–150 g body weight; Charles River Laboratories, Wilmington, MA). All animal experiments were performed with the approval of the Medical College of Wisconsin Institutional Animal Care and Use Committee in accordance with the National Institutes of Health Guidelines for the Care and Use of Laboratory. The estimated numbers of animals needed were derived from our previous experience with similar experiments,^26,58^ and power analysis was not performed. The numbers of rats used were detailed in the relevant sections or figure legends.

### 2.2. Induction of knee osteoarthritis

As previously described, the MIA model of knee OA (right knee) was induced during isoflurane-anesthesia via intra-articular cavity injection of 2 mg MIA (Sigma-Aldrich, St. Louis, MO) in 50 μL sterile PBS.^26,55^ Control rats received an intra-articular injection of saline (50 μL).

### 2.3. Behavior tests

Behavioral tests were conducted between 9:00 am and 12:00 pm. Experimenters were blinded to the vector treatment in MIA rats. Stimulated sensory behavior tests, including nonnoxious mechanical allodynia (von Frey [vF]), noxious mechanical hyperalgesia (Pin test), cooling stimulation (cold), heating plantar test (heat), and spontaneous pain behavior tests, including conditioned place preference (CPP) and static weight-bearing asymmetry (Wb) using an Incapacitance meter, were described previously.^7,13^

### 2.4. Adeno-associated virus constructs

Adeno-associated virus vectors were packed into serotype 6.2FF.^65^ Serotype 6.2FF was engineered by mutagenesis (Genscript, Piscataway, NJ) to generate a triple-mutant AAV6 capsid encoding F129L, Y445F, and Y731F point mutations.^65^ Production, purification, and titration of AAVs, including AAV6.2FF-(green fluorescent protein) GFP-Na_V_iPA1 (AAV6.2FF-Na_V_iPA1) and AAV6.2FF-GFP-NP (AAV6.2FF-NP, NP (N-terminal peptide) is an inert peptide from Na_V_1.7 protein N-terminus as control), have been described previously.^57^ The titers (GC/mL) of AAV6.2FF-Na_V_iPA1 and AAV6.2FF-NP were 2.05 × 10^13^ and 2.34 × 10^13^, respectively. One lot of viral preparation for each vector was used for in vivo experiments.

### 2.5. Microinjection of adeno-associated virus vectors into DRG

Adeno-associated virus vector was microinjected into the right lumbar (L) 4 and L5 dorsal root ganglia (DRG) using previously described techniques,^13,26,57,58^ employing a microprocessor-controlled injector (Nanoliter 2000, World Precision Instruments, Sarasota, FL).^13,26,57,58^ Rats received L4 and L5 DRG injections of either AAV6.2FF-Na_V_iPA1 or AAV6.2FF-NP (1 vector per rat), consisting of 2 μL with adjusted titers containing a total of 2.0 × 10^10^ viral particles for each DRG.

### 2.6. Histology and immunohistochemistry

#### 2.6.1. Knee histopathological analysis

The knee joints (distal femur to proximal tibia) were resected and fixed with 10% neutral-buffered formalin for 1 week at room temperature. The fixed specimens were decalcified in Immunocal (ThermoFisher, Rockford, IL) for 2 weeks and embedded in paraffin. The rehydrated paraffin sections were stained with hematoxylin and eosin (H&E), as described previously.^55^ Images of knee joint histology were captured using a Keyence BZ-X800 microscope (Keyence Corporation, Itasca, IL).

#### 2.6.2. Immunohistochemistry and immunohistochemistry quantification

Our previously described protocol was adopted.^57,58^ The formalin-fixed, paraffin-embedded tissue sections were used. Primary antibodies: mouse GFP (1:500, Santa Cruz Biotechnology, SCB, CA. sc9996), rabbit GFP (1:500, Cell Signaling, Danvers, MA. 2555), rabbit Na_V_1.7 (1:400, Alomone, Jerusalem, Israel. ASC008), rabbit Na_V_1.6 (1:400, Alomone, ASC009), rabbit glial fibrillary acidic protein (GFAP, 1:1000, Dako, CA, Z0334), mouse NF200 (1:1000, Sigma-Aldrich, N5389), and mouse β3Tubulin (Tubb3, 1:500, SCB, sc-80016). The appropriate fluorophore-conjugated (Alexa 488 or Alexa 594, 1:2000) secondary antibodies (Jackson ImmunoResearch, West Grove, PA) were used to reveal immune complexes. Normal immunoglobulin G (IgG from the same species as the first antibody) was replaced for the first antibody as the negative control (Suppl. Fig. 2 and suppl. method, http://links.lww.com/PR9/A316). Immunohistochemistry (IHC) quantification was performed as we previously described.^57,58,69^

### 2.7. Whole-cell current-clamp recording of dissociated DRG neurons

Dissociated DRG neuronal culture for electrophysiology was performed as described previously,^58^ and dissociated neurons were studied in 6 to 8 hours after harvest in electrophysiological experiments.

Whole-cell current-clamp recording was performed, as described previously^58^ (Suppl. Method, http://links.lww.com/PR9/A316), to determine the effects of AAV-mediated Na_V_iPA1 expression on neuronal excitability. Dissociated small- and medium-sized DRG neurons (<35 μm in diameter) from saline-injected animals and rats with MIA only and dissociated DRG neurons with clear GFP expression from MIA rats injected with AAV6.2FF-NP or AAV6.2FF-Na_V_iPA1at 6 weeks after vector injection were used for recording (n = 4∼5 rats per group). For whole-cell current-clamp, patch electrodes had a resistance of 0.7 to 1.5 MΩ when filled with the pipette solution. The pipette and bath solution were prepared as described previously.^57,58^ Action potentials (APs) were generated by injection of a series of current pulses (100–1000 pA in steps of 100 pA, 300 ms). The baseline membrane potential was recorded for 20 milliseconds before the stimulus pulses were injected into the neurons. We defined the resting membrane potential (RMP) as the mean value of the 20-millisecond prestimulus membrane potential in the first trial and the AP rheobase as the minimum depolarizing current required to evoke the first AP. The neurons with stable RMP more negative than −45 mV and overshooting APs (>80 mV RMP to peak) were used for additional data collection. Action potential frequency, an indicator of PSN excitability, was determined by quantifying the number of APs elicited in response to depolarizing current injections (300 ms).

### 2.8. Statistical analysis

All data are presented as mean ± standard error of the mean (SEM) and were analyzed with GraphPad PRISM 10 (GraphPad Software, San Diego, CA). A priori level of significance at 95% confidence level was considered at *P* < 0.05. Behavioral changes were analyzed by 1-way or 2-way ANOVA followed by turkey post hoc and student *t*-test, where appropriate, as described previously.^57^ The differences in the electrophysiological experiments were compared with 1-way ANOVA and turkey post hoc.

## 3. Results

### 3.1. Monoiodoacetate-induced knee damages after adeno-associated virus treatment in the current study were overall comparable to those in our previous reports

Monoiodoacetate-treated knees in both male and female animals displayed typical structural joint damage, examined 56 days after MIA knee cavity injection and 42 days after AAV6.2FF-Na_V_iPA1 treatment.^26,55^ Knees injected with 2 mg of MIA showed a considerable loss of articular cartilage surrounding the subchondral bone along the joint, combined with reduced chondrocyte numbers, variable degrees of bone marrow lesions, and collapse of subchondral bone (Suppl. Fig. 1, http://links.lww.com/PR9/A316). This study did not attempt to correlate MIA-induced cartilage loss, as well as other knee degeneration pathology, to pain behaviors, as the study design was focused on determining if the sensory neuron–selective block of multiple TTXs Na_V_s by Na_V_iPA1 will mitigate neuropathic pain–like behaviors.

### 3.2. Analgesia of monoiodoacetate-osteoarthritis pain by DRG delivery of adeno-associated virus-Na_V_iPA1 in male rats

**Figure 1.**
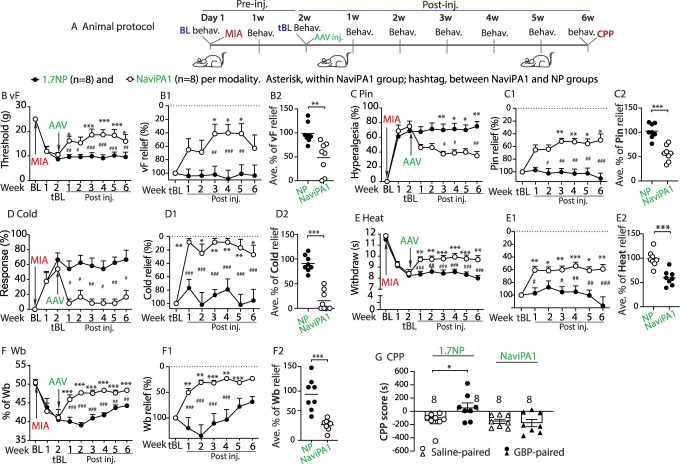
Analgesia of established MIA-OA pain in male rats. An animal protocol is schematically outlined (A). Time courses of vF, Pin, Heat, Cold, and Wb before and after treatment (B–F); *P < 0.05, **P < 0.01, and ***P < 0.001 for comparisons to tBL within Na_V_iPA1-treated group and #*P* < 0.05, ##*P* < 0.01, and ###*P* < 0.001 for comparisons between Na_V_iPA1 and NP groups. Repeated measures parametric 2-way ANOVA for vF, Heat, and Wb followed by Tukey post hoc; nonparametric Friedman ANOVA for Pin and Cold tests and Dunn post hoc. The measures on the 14th day after MIA and before AAV treatment (tBL) were converted as the peak pain intensity (100%) and the measures of each sensory modality after treatment were normalized to tBL measures and the percentage of pain relief for each modality at multiple time points was calculated (B1-F1). *P < 0.05, **P < 0.01, and ***P < 0.001 for comparisons to the tBL within Na_V_iPA1-treated group and #*P* < 0.05, ##*P* < 0.01, and ###*P* < 0.001 between groups. Repeated measures 2-way ANOVA for vF and Heat, and Tukey (within Na_V_iPA1group) and Bonferroni (between Na_V_iPA1 and NP groups) post hoc; nonparametric Friedman ANOVA for Pin and Cold tests and Dunn post hoc. Summed average pain relief in 6-week treatment showed 52%, 45%, 82%, 40%, and 40% reduction of vF-, Pin-, Cold-, and Heat-stimulated hypersensitivity, respectively (B2-F2). **P < 0.01 and ***P < 0.001, unpaired, 2-tailed Student *t*-test. (G) CPP scores (seconds, s) of saline-paired chamber and the GBP-paired chamber between AAV-Na_V_iPA1 or AAV-NP, *P < 0.05 (unpaired, 2-tailed Student *t*-test). AAV, adeno-associated virus; CPP, conditioned place preference; GBP, gabapentin; MIA, monoiodoacetate; NP, N-terminal peptide; OA, osteoarthritis; tBL, treatment baseline.

Na_V_iPA1 is a multipronged Na_V_ inhibitory peptide that blocks the activity of TTXs Na_V_1.7, Na_V_1.6, Na_V_1.3, and Na_V_1.1, which is derived from Na_V_1.7 intrinsically disordered domain that is highly conserved within the intracellular loop 1 of TTXs Na_Vs_.^57^ Our prior data showed that AAV-mediated, PSN-specific expression of Na_V_iPA1 is effective in relieving pain behaviors in tibial nerve injury (TNI) rats without affecting normal pain thresholds in naïve animals.^57^ To broaden the testing of analgesic effects across different pain models, we set up experiments to examine whether DRG delivery of AAV6.2FF-Na_V_iPA1 is also effective in mitigating MIA-OA pain behaviors. In the experimental design (Fig. 1A), the sensitivity to mechanical and thermal hind paw cutaneous stimulation as well as asymmetric hind limb weight bearings on the arthritic limb loading were assessed at baseline (before MIA) and weekly after MIA knee injection for 2 weeks before DRG delivery of AAV6.2FF-Na_V_iPA1 and AAV6.2FF-NP (control). After behavior tests on the 14th day after MIA-OA, rats were randomized to receive injection of either AAV6.2FF-Na_V_iPA1 or AAV6.2FF-NP into the ipsilateral L4/L5 DRG, since these DRG innervate knee and sciatic nerves and show evidence of injury in knee MA-OA animals.^27,55^ Thereafter, sensory behaviors and weight-bearing asymmetry were evaluated weekly for additional 6 weeks. As a terminal experiment, gabapentin (GBP)-induced CPP was performed in both groups after the observation course to evaluate the affective element of spontaneous pain between treatment and control groups. After this, the tissues were harvested for IHC characterization of transgene and target gene expression and for current-clamp recordings of PSN AP firing. Sensory behavior measures before vector injection on the 14th day after MIA knee injection were used as a treatment baseline (tBL) to compare the effects of vector injection.

Results showed that all rats receiving 2-mg MIA knee injection developed marked ipsilateral pain behaviors, tested on the plantar surface of the arthritic hind paw, which included reduced threshold for withdrawals from mild mechanical stimuli (vF), more frequent hyperalgesic-type responses after noxious mechanical stimulation (Pin), more frequent withdrawals to heat and cold (acetone) stimuli, and increased arthritic hind limb weight-bearing asymmetry. These behaviors persisted in arthritic hind limbs after injection of the control AAV6.2FF-NP during the 6-week postinjection observation. In contrast, MIA rats injected with AAV6.2FF-Na_V_iPA1 showed progressive and persistent reversal of these behavioral sensitizations (Figs. 1B–F). Monoiodoacetate-induced hind limb asymmetry of weight distribution in the arthritic limbs paralleled changes in the ipsilateral hind paw mechanical and thermal sensitivity, suggesting neuropathic components responsible for weight-bearing asymmetry after 2 weeks of inflammation stage.^26^ The typical requirement for a “minimum clinically difference” in analgesic trials is >30% reduction of pain indicators.^12,36,38^ Here in our preclinical study, behavior testing on the 14th day after MIA was normalized against the peak possible measure (100%) for each animal pain test, and compared to the measures at the tBL, to calculate the percentage of pain behavior reduction in the 6-week treatment course (Figs. 1B1–F1). Results showed an average reduction (%) of 52, 45, 82, 40, and 40 for vF-, Pin-, Cold-, and Heat-stimulated mechanical and thermal pain behaviors, and weight-bearing asymmetry, respectively (Figs. 1B2–F2).

Spontaneous pain is a feature of knee MIA-OA pain and CPP is a method of testing the presence of the affective aspect of spontaneous pain.^26,43^ Using a biased CPP paradigm,^26,46^ the effect of AAV6.2FF-Na_V_iPA1 and control AAV6.2FF-NP treatment on spontaneous pain was evaluated. None of the animals in both groups were excluded from the study because of their extreme baseline preference/avoidance for a chamber.^26^ A significant CPP in response to GBP was observed in the MIA rats injected with AAV6.2FF-NP, indicating ongoing pain that is sensitive to GBP, consistent with previous findings.^20^ However, there was no significant difference in the times spent in the initially nonpreferred chamber during baseline vs test period in AAV6.2FF-Na_V_iPA1-treated animals (Fig. 1G), indicating the absence of ongoing pain in these animals and AAV6.2FF-Na_V_iPA1 relieves ongoing pain caused by prior MIA.

### 3.3. Adeno-associated virus-mediated Na_V_iPA1 transduction in vivo

**Figure 2.**
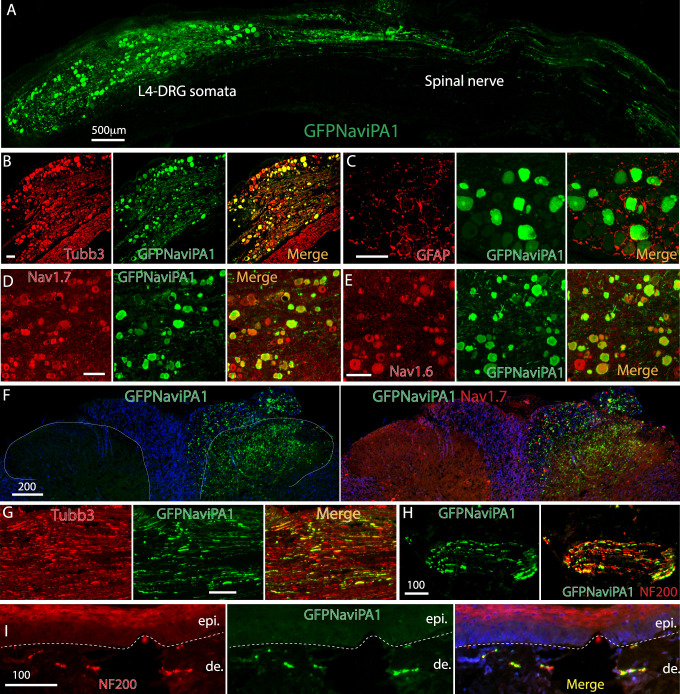
IHC of GFP-Na_V_iPA1 and target gene expression. Illustrations show representative GFP-Na_V_iPA1 expression in DRG neurons and their central and peripheral processes 6 weeks after AAV6-Na_V_iPA1 injection. (A) A large IHC image of GFP-Na_V_iPA1 shows expression profile in L5-DRG somata and along its spinal nerve. (B and C) IHC montage images show colocalization of GFP-Na_V_iPA1 (green) with Tubb3-positive neurons (red) (B) but not with GFAP-positive perineuronal glia (red) (C). (D and E) IHC montage images show colocalization of GFP-Na_V_iPA1 (green) with Nav1.7-positive (red) (D) and Nav1.6-positive neurons (red) (E). (F) IHC montage images show GFPNa_V_iPA1 (green) and Na_V_1.7 (red) in PSN central terminals innervating spinal dorsal horn. (G–I) IHC montage images illustrate GFPNa_V_iPA1 (green), colabeled with Tubb3 (red) in sciatic nerve (G) and with NF200 (red) in PSN peripheral terminals (H) and hind paw skin (I). Scale bar (μm): (A) 500; (B–E) 100; (F) 200; (G–I) 100. AAV, adeno-associated virus; DRG, dorsal root ganglia; GFAP, glial fibrillary acidic protein; GFP, green fluorescent protein; IHC, Immunohistochemistry; PSN, peripheral sensory neuron.

The in vivo transduction rate of AAV6.2FF-Na_V_iPA1 6 weeks after vector injection was determined by IHC. The Na_V_iPA1-positive neurons (GFP) comprised 57 ± 15% (1066 out of 1924 total neuronal profiles, positive for pan-neuronal marker Tubb3, n = 3 DRG [all L4 from 3 rats, 3–4 sections per DRG, which were selected as every fifth section from the consecutive serial sections]). Transduced DRG neurons included the full-size range of the PSNs (Figs. 2A–E) that also expressed Tubb3, Na_V_1.7, and Na_V_1.6 (Figs. 2D and E), but GFP-Na_V_iPA1 signals were not colabeled in the GFAP-positive DRG perineuronal glia cells (Fig. 2C). In addition, GFP-Na_V_iPA1 signals were extensively detected in the afferent central terminals innervating the spinal dorsal horn, axons in sciatic nerve, and PSN peripheral terminals innervating hind paw skin, ipsilateral to DRG injection (Figs. 2F–I).

### 3.4. Reversal of knee monoiodoacetate-induced peripheral sensory neuron hyperexcitability by adeno-associated virus-Na_V_iPA1 treatment (male rats)

**Figure 3.**
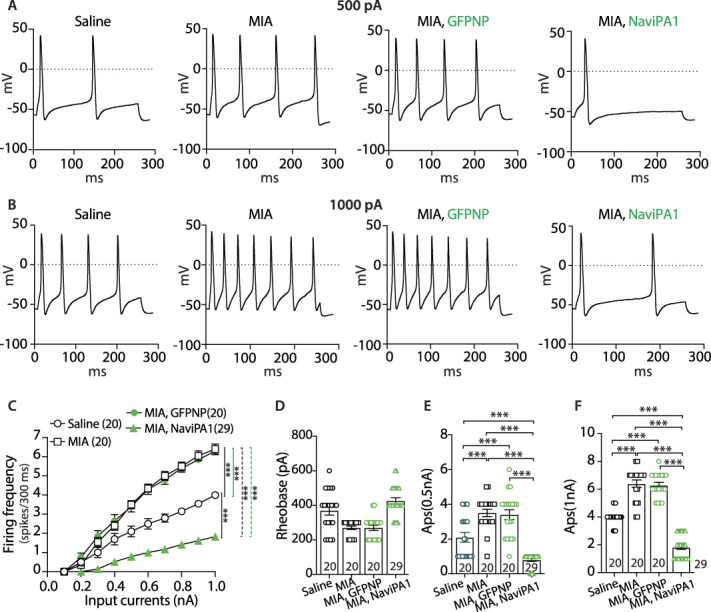
Current-clamp recordings of Na_V_iPA1 expression on PSN excitability (male). Representative AP traces elicited by 300 milliseconds depolarizing current of 500 pA (A) and 1000 pA (B) (same cells) from resting membrane potential (RMP) were recorded on PSNs dissociated from the rats of saline, MIA only, and GFP-expressing neurons in MIA rats treated with AAV-Na_V_iPA1 or AAV-NP, as indicated. Comparison of responses (number of APs evoked by a 300 ms stimulus) for the PSNs in different groups across a range of step current injections from 100 to 1000 pA (C); ***P < 0.001, 2-way ANOVA of main effects of the groups with Bonferroni post hoc. Scatter plots with bars show the rheobases (D) and AP numbers evoked by input current at 500 pA (E) and 1000 pA (F) from RMP, respectively. The number in each group is the number of analyzed neurons per group. ***<0.001, 1-way ANOVA and Turkey post hoc. AAV, adeno-associated virus; AP, action potential; GFP, green fluorescent protein; MIA, monoiodoacetate; NP, N-terminal peptide; PSN, peripheral sensory neuron.

Na_V_ channels contribute to nociception by driving burst firing of AP in PSNs,^11^ injury-induced PSN hyperexcitability results in pain,^19^ and inhibition of Na_V_ channels in sensory neurons presents a promising novel modality for the treatment of pain.^2^ Our previous study showed that Na_V_iPA1 attenuation of neuropathic pain in a TNI model is associated with the reversal of injury-induced neuronal hyperexcitability.^57^ We next test whether analgesia of DRG-Na_V_iPA1 treatment in MIA-OA pain is associated with suppression of PSN hyperexcitability. Although MIA results in DRG with comingled injured and uninjured axons, nerve injury can induce an increase of voltage-gated ion channel activity in both injured neurons and adjacent intact neurons, leading to similar electrophysiological changes and increased discharge frequency in damaged and neighboring intact DRG neurons.^30^ We, therefore, recorded from randomly chosen small- and medium-sized neurons in the cultures from dissociated L4 DRG. Sensory neurons (small/medium, <35 μm in diameter)^56^ dissociated from DRG of saline-injected rats and MIA rats without treatment, GFP-expressing neurons injected with either AAV6.2FF-NP and AAV6.2FF- Na_V_iPA1, were used for recording. Transduced neurons were identified by GFP fluorescence, and excitability was evaluated by measuring the rheobase and the repetitive firing during 300-millisecond current injection steps. The effect of Na_V_iPA1 expression on the DRG-PSN repetitive firing properties was assessed by applying a series of 300-millisecond current injections to the DRG dissociated neurons. Although RMP in the neurons from MIA did not differ from saline animals as reported previously,^22^ the frequency of APs evoked by progressively greater depolarizations in the recorded neurons from MIA rats was significantly increased, compared to saline controls, as previously reported.^26,59^ The increased PSN excitability in MIA rats was reversed in the transduced neurons after AAV6.2FF-Na_V_iPA1 treatment, whereas AAV6.2FF-NP-transduced neurons had no effect (Fig. 3). Together, these findings indicate that the reversal of MIA-induced sensory neuronal hyperexcitability^55^ by Na_V_iPA1 contributes to its attenuation of neuropathic pain behaviors.

### 3.5. Analgesia of DRG adeno-associated virus-Na_V_iPA1 treatment in female monoiodoacetate-osteoarthritis rats

**Figure 4.**
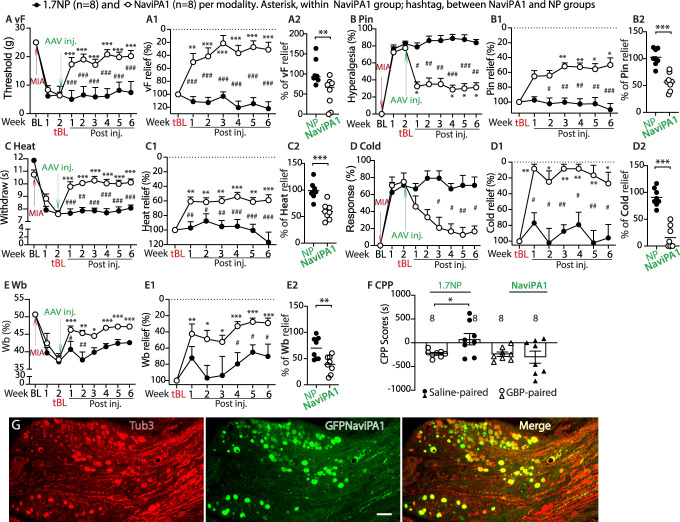
Analgesia of DRG-AAV-Na_V_iPA1 injection in female MIA rats. Analogous figures to Figure 1 show analgesia after AAV-Na_V_iPA1 treatment to the established MIA pain in female rats. *P < 0.05, **P < 0.01, and ***P < 0.001 for comparisons to the treatment baseline (tBL) within Na_V_iPA1-treated group and #*P* < 0.05, ##*P* < 0.01, and ###*P* < 0.001 for comparisons between Na_V_iPA1 and NP groups (A–E). The tBL measures were converted as the peak pain intensity (100%) and the measures of each sensory modality after treatment were normalized to the measures of tBL and the percentage of pain relief for each modality at multiple time points was calculated (A1-E1). *P < 0.05, **P < 0.01, and ***P < 0.001 for comparisons to the tBL within Na_V_iPA1-treated group and #*P* < 0.05, ##*P* < 0.01, and ###*P* < 0.001 between Na_V_iPA1 and NP groups. Repeated measures 2-way ANOVA for vF and Heat, and Tukey (within Na_V_iPA1 group) and Bonferroni (between Na_V_iPA1 and NP groups) post hoc; nonparametric Friedman ANOVA for Pin and Cold tests and Dunn post hoc. Summed average pain relief in 6-week treatment showed 44%, 45%, 82%, 40%, and 44% reduction of vF-, Pin-, Cold-, and Heat-stimulated hypersensitivity, respectively (A2-E2). **P < 0.01 and ***P < 0.001, unpaired, 2-tailed Student *t*-test. CPP scores (s) of saline-paired chamber and GBP-paired chamber between AAV-Na_V_iPA1 and AAV-NP, **P < 0.01 (unpaired, 2-tailed Student *t* test) (F). Representative IHC montage images show expression profile of GFPNa_V_iPA1 after AAV injection in a female MIA rat (6 weeks) (G, G1). Scale bar: 500 μm. AAV, adeno-associated virus; CPP, conditioned place preference; GBP, gabapentin; GFP, green fluorescent protein; IHC, Immunohistochemistry; MIA, monoiodoacetate; NP, N-terminal peptide.

Sex differences exist in experimental and clinical pain and in response to pain interventions.^41^ We, therefore, next tested whether DRG-AAV6.2FF-Na_V_iPA1 treatment is also effective in attenuating pain behaviors and weight-bearing asymmetry induced by MIA knee injection in female animals, using the same animal protocol as that in male rats (Fig. 2A). Specifically, vector injection was performed 2 weeks after right knee MIA, both evoked mechanical and thermal nociception, arthritic limb weight-bearing asymmetry, and GBP-CPP 6 weeks after AAV treatments were evaluated. The same batch AAV preparation used in the male rats was applied. Results (Figs. 4A–F) showed that the female MIA-OA rats displayed phenotypic development of hypersensitivity after induction of MIA-OA comparable to male rats and that both evoked mechanical/thermal hypersensitivity, arthritic limb weight-bearing asymmetry, and GBP-CPP responses after 6-week treatment were substantially normalized after AAV6.2FF-Na_V_iPA1 treatment, showing analgesic effects comparable to those in male animals. The average reduction (%) of vF-, Pin-, Cold-, and Heat-stimulated mechanical and thermal pain behaviors and arthritic limb weight-bearing asymmetry in the 6-week treatment course was 44, 45, 82, 40, and 44, respectively. Immunohistochemistry on the DRG sections from the female MIA rats 6 weeks after intraganglia injection of AAV6.2FF-Na_V_iPA1 also revealed a comparable GFP-Na_V_iPA1 expressive profile to the male rats (Figs. 4G and G1). In vivo transduction rate is 53 ± 16% (479 out of 969 total neuronal profiles, positive for Tubb3). Thus, although male and female groups were not directly compared, sexual dimorphism was not apparent for either pain behavior phenotypes after MIA or in the response to DRG-AAV6.2FF-Na_V_iPA1 treatment in this study.

Together, these findings suggest that AAV-mediated, PSN-specific Na_V_iPA1 treatment has clear analgesic efficacy in normalizing the established peripheral hypersensitivity displaying as evoked and spontaneous ongoing pain in rat MIA-induced OA-pain, effective in both males and females. In addition, the arthritic limb weight-bearing asymmetry was also significantly normalized. A sexual dimorphism seemed not apparent for both pain behavior phenotypes after MIA knee injection and in responsivity to DRG-AAV6.2FF-Na_V_iPA1 treatment. Notably, the sustained analgesic effectiveness after treatment was maintained for several weeks and predicted to outlast the observation period. Long-term analgesic effectiveness will require evaluation in future studies.

## 4. Discussion

Targeted PSN analgesia without risk of addiction is an unmet medical need.^45^ Our data affirm that AAV6.2FF-mediated, PSN-selective block of TTXs-Na_V_ by Na_V_iPA1 significantly mitigates hind paw evoked hypersensitivity and normalizes the arthritic limb weight-bearing asymmetry and the aversiveness of ongoing pain of knee MIA-OA in rats. These data suggest that PSN abnormality in sensory neuron somata is a significant driver for MIA-OA pain. Dysfunction of Na_V_ channels in PSNs appears to be a pathological requisite that contributes to the development and maintenance of neuropathic pain–like behaviors in MIA-OA,^40^ and block of the firing of APs through inhibition of PSNs-Na_V_s may be an effective means of mediating analgesia in MIA-OA. In addition, the inhibitory effects of Na_V_iPA1 to TTXs-Na_V_s are reproducible in the hiPSC-SNs,^57^ indicating a translational potential. Since multiple subtypes of sodium channels contribute to nociceptive electrogenesis,^1,6^ our results suggest that block of several of these selective in DRG-PSNs might be a therapeutical advantage.

Osteoarthritis chronic pain can be generated and maintained at different levels along the neuraxis, all of which may provide the opportunity for pharmacological intervention.^33^ In the early OA stage, pain generation is conceivably initiated at the injury site due to sensitized PSNs innervating joint, by which the specialized sensory afferents detect joint damage-derived chemical, mechanical, or thermal algogenic stimuli. This justifies the local intervention of sensitized sensory afferents, ie, pain arising from the site of injury.^3,29,35,54^ A recent report shows that functional Na_V_1.7 is expressed in chondrocytes and demonstrates that Na_V_1.7 is a chondrocyte regulator and therapeutic target for osteoarthritis.^16^ In addition to pathology at the arthritic joint, OA also progressively induces morbidity in the PSN somata,^32,44^ especially at the advanced stage of the disease.^21^ More recent literature has documented profound molecular and cellular changes in the DRG of various OA models, including alterations in neuronal excitability,^50^ intense proliferation and activation of microglia that mediate neuroinflammation and neuronal damage,^31,42,64,70^ transcriptomic upregulation of the genes for neuroimmune interactions,^39^ sensitized PSN nociceptive voltage-gated ion channels^16,51,55^ and mechanosensitive ion channels,^22^ and upregulated chemokine and cytokine production,^18^ all contributing interrelatedly in driving continuous neural sensitization after cessation of joint inflammation.^60,66^

Several mechanisms may dictate that DRG-PSN morbidity in OA is not limited exclusively to knee-innervating PSNs. Primarily, although PSN somata in DRG are anatomically isolated from each other and are not synaptically interconnected, most DRG-PSNs are transiently depolarized when axons of neighboring neurons of the same ganglion are stimulated repetitively.^4^ This implies that peripheral tissue inflammation and sensitization can induce DRG neuronal cross-depolarization coupling that adjacent neurons activate, together contributing to pain hypersensitivity.^30^ This coupling activation occurs among various sized neurons including small-diameter nociceptors and large-diameter low-threshold mechanoreceptors.^30^ Therefore, although MIA produces DRG with comingled sensitized with “normal” axons, damaged neurons can induce an increase of pronociceptive ion channel activity in both damaged neurons and adjacent intact neurons, leading to similar electrophysiological changes and increased discharge frequency in the damaged and neighboring intact PSNs.^9^ In addition, microglial mechanisms contribute to OA-pain as in neuropathic pain,^17^ and the activated microglia can sense and regulate neuronal activity, which is expected to affect all populations of PSNs since the highly proliferated microglia can be detected in wrapping and close contact with the PSN cell bodies in DRG ipsilateral to MIA injection,^52,55^ which play a significant role in the maintenance of mechanical hypersensitivity in inflammatory arthritis.^52^ Furthermore, increased nerve growth factor (NGF) and tumor necrosis factor-α (TNF-α) are common pathological mediators in both neuropathic pain and OA pain, and both are known determinants in regulating the neuronal excitability via upregulation of multiple Na_V_s, including Na_V_1.7, Na_V_1.3, Na_V_1.6, and Na_V_1.8 that can involve in both injured and uninjured DRG neurons after nerve injury.^5,16,53^

Our results proved that AAV-mediated DRG-targeted block of PSN TTXs-Na_V_s succeeded in attenuating neuropathic pain behaviors in a rat model of MIA-OA pain. For clinical analgesia, the peripheral sensory nervous system (PSNS) is a particularly accessible site for devising new treatments, especially the DRG-PSNs that initiate nociception and have a central role in the development and maintenance of painful neuropathy.^23,37^ Delivering drugs to PSNs is well-developed and safe, for instance, as used in clinical anesthesia for regional blockade and by pain physicians for diagnosis and treatment of radiculopathy.^47^ Injection into the DRG has minimal consequences in preclinical models^13^ and unintentional intraganglion injection commonly accompanies clinical foraminal epidural steroid injection,^47^ a very common procedure with minimal risk of nerve damage. Our findings, coupled with known toxicity related to systemic Na_V_ antagonism (ie, CNS and heart), have set the premise that AAV-mediated, PSN-specific block of multiple nociceptive Na_V_s could be developed to treat intractable OA pain conditions that are difficult to control with standard medical care.

This study is formulated to test the effectiveness of targeting DRG TTXs-Na_V_s for OA-pain. A limitation is that a MIA-OA model can be differentiated in pain pathogenesis from human OA-pain; however, features of MIA-induced disease are relevant to some aspects of human OA,^48^ including the knee damages and neuropathic pain–like symptoms. In addition, whether DRG-targeted approach would prevent the nociceptive signals from reaching the spinal cord dorsal horn will be further investigated. Recent studies report that central nervous system gene therapy by intravenous high-dose AAV causes asymptomatic and self-limited DRG inflammation and mild PSN degeneration in primates.^8,24^ Since these changes are very minor in comparison to those induced by painful inflammatory and neuropathic conditions that AAV injection would treat, this is unlikely to become a barrier to the clinical application of our approach.

## Disclosures

The authors declare that they have no known competing financial interests or personal relationships that could have appeared to influence the work reported in this paper.

## Supplemental digital content

Supplemental digital content associated with this article can be found online at http://links.lww.com/PR9/A316.

## Supplementary Material

SUPPLEMENTARY MATERIAL

## References

[1] Alsaloum M Higerd GP Effraim PR Waxman SG. Status of peripheral sodium channel blockers for non-addictive pain treatment. Nat Rev Neurol 2020;16:689–705. 33110213 10.1038/s41582-020-00415-2

[2] Alsaloum M Labau JIR Liu S Estacion M Zhao P Dib-Hajj F Waxman SG. Contributions of Na(V)1.8 and Na(V)1.9 to excitability in human induced pluripotent stem-cell derived somatosensory neurons. Sci Rep 2021;11:24283. 34930944 10.1038/s41598-021-03608-x PMC8688473

[3] Alves CJ Couto M Sousa DM Magalhães A Neto E Leitão L Conceição F Monteiro AC Ribeiro-da-Silva M Lamghari M. Nociceptive mechanisms driving pain in a post-traumatic osteoarthritis mouse model. Sci Rep 2020;10:15271. 32943744 10.1038/s41598-020-72227-9 PMC7499425

[4] Amir R Devor M. Chemically mediated cross-excitation in rat dorsal root ganglia. J Neurosci 1996;16:4733–41. 8764660 10.1523/JNEUROSCI.16-15-04733.1996 PMC6579034

[5] Barker PA Mantyh P Arendt-Nielsen L Viktrup L Tive L. Nerve growth factor signaling and its contribution to pain. J Pain Res 2020;13:1223–41. 32547184 10.2147/JPR.S247472 PMC7266393

[6] Bennett DL Clark AJ Huang J Waxman SG Dib-Hajj SD. The role of voltage-gated sodium channels in pain signaling. Physiol Rev 2019;99:1079–151. 30672368 10.1152/physrev.00052.2017

[7] Bove SE Calcaterra SL Brooker RM Huber CM Guzman RE Juneau PL Schrier DJ Kilgore KS. Weight bearing as a measure of disease progression and efficacy of anti-inflammatory compounds in a model of monosodium iodoacetate-induced osteoarthritis. Osteoarthritis Cartilage 2003;11:821–30. 14609535 10.1016/s1063-4584(03)00163-8

[8] Buss N Lanigan L Zeller J Cissell D Metea M Adams E Higgins M Kim KH Budzynski E Yang L Liu Y Butt M Danos O Fiscella M. Characterization of AAV-Mediated dorsal root ganglionopathy. Mol Ther Methods Clin Dev 2022;24:342–54. 35229008 10.1016/j.omtm.2022.01.013 PMC8851102

[9] Campbell JN Meyer RA. Mechanisms of neuropathic pain. Neuron 2006;52:77–92. 17015228 10.1016/j.neuron.2006.09.021 PMC1810425

[10] Dib-Hajj SD Waxman SG. Disordered but effective: short linear motifs as gene therapy targets for hyperexcitability disorders. J Clin Invest 2024;134:e182198. 38949022 10.1172/JCI182198 PMC11213459

[11] Dib-Hajj SD Yang Y Black JA Waxman SG. The Na(V)1.7 sodium channel: from molecule to man. Nat Rev Neurosci 2013;14:49–62. 23232607 10.1038/nrn3404

[12] Farrar JT Berlin JA Strom BL. Clinically important changes in acute pain outcome measures: a validation study. J Pain Symptom Manage 2003;25:406–11. 12727037 10.1016/s0885-3924(03)00162-3

[13] Fischer G Kostic S Nakai H Park F Sapunar D Yu H Hogan Q. Direct injection into the dorsal root ganglion: technical, behavioral, and histological observations. J Neurosci Methods 2011;199:43–55. 21540055 10.1016/j.jneumeth.2011.04.021 PMC3742008

[14] French HP Smart KM Doyle F. Prevalence of neuropathic pain in knee or hip osteoarthritis: a systematic review and meta-analysis. Semin Arthritis Rheum 2017;47:1–8. 28320529 10.1016/j.semarthrit.2017.02.008

[15] Fu K Robbins SR McDougall JJ. Osteoarthritis: the genesis of pain. Rheumatology (Oxford) 2018;57:iv43-50. 29267879 10.1093/rheumatology/kex419

[16] Fu W Vasylyev D Bi Y Zhang M Sun G Khleborodova A Huang G Zhao L Zhou R Li Y Liu S Cai X He W Cui M Zhao X Hettinghouse A Good J Kim E Strauss E Leucht P Schwarzkopf R Guo EX Samuels J Hu W Attur M Waxman SG Liu CJ. Na(v)1.7 as a chondrocyte regulator and therapeutic target for osteoarthritis. Nature 2024;625:557–65. 38172636 10.1038/s41586-023-06888-7 PMC10794151

[17] Geraghty T Winter DR Miller RJ Miller RE Malfait AM. Neuroimmune interactions and osteoarthritis pain: focus on macrophages. Pain Rep 2021;6:e892. 33981927 10.1097/PR9.0000000000000892 PMC8108586

[18] Gonçalves WA Rezende BM de Oliveira MPE Ribeiro LS Fattori V da Silva WN Prazeres P Queiroz-Junior CM Santana KTdO Costa WC Beltrami VA Costa VV Birbrair A Verri WA Jr Lopes F Cunha TM Teixeira MM Amaral FA Pinho V. Sensory ganglia-specific TNF expression is associated with persistent nociception after resolution of inflammation. Front Immunol 2019;10:3120. 32038637 10.3389/fimmu.2019.03120 PMC6984351

[19] Haroutounian S Nikolajsen L Bendtsen TF Finnerup NB Kristensen AD Hasselstrøm JB Jensen TS. Primary afferent input critical for maintaining spontaneous pain in peripheral neuropathy. PAIN 2014;155:1272–9. 24704366 10.1016/j.pain.2014.03.022

[20] Havelin J Imbert I Cormier J Allen J Porreca F King T. Central sensitization and neuropathic features of ongoing pain in a rat model of advanced osteoarthritis. J Pain 2016;17:374–82. 26694132 10.1016/j.jpain.2015.12.001 PMC4824638

[21] Haywood AR Hathway GJ Chapman V. Differential contributions of peripheral and central mechanisms to pain in a rodent model of osteoarthritis. Sci Rep 2018;8:7122. 29740093 10.1038/s41598-018-25581-8 PMC5940779

[22] He BH Christin M Mouchbahani-Constance S Davidova A Sharif-Naeini R. Mechanosensitive ion channels in articular nociceptors drive mechanical allodynia in osteoarthritis. Osteoarthritis Cartilage 2017;25:2091–9. 28882752 10.1016/j.joca.2017.08.012

[23] Hogan QH. Labat lecture: the primary sensory neuron: where it is, what it does, and why it matters. Reg Anesth Pain Med 2010;35:306–11. 20460965 10.1097/AAP.0b013e3181d2375e PMC2885292

[24] Hordeaux J Buza EL Dyer C Goode T Mitchell TW Richman L Denton N Hinderer C Katz N Schmid R Miller R Choudhury GR Horiuchi M Nambiar K Yan H Li M Wilson JM. Adeno-associated virus-induced dorsal root ganglion pathology. Hum Gene Ther 2020;31:808–18. 32845779 10.1089/hum.2020.167

[25] Hunter DJ McDougall JJ Keefe FJ. The symptoms of osteoarthritis and the genesis of pain. Med Clin North Am 2009;93:83–100, xi. 19059023 10.1016/j.mcna.2008.08.008

[26] Itson-Zoske B Shin SM Xu H Qiu C Fan F Hogan QH Yu H. Selective block of sensory neuronal T-type/Cav3.2 activity mitigates neuropathic pain behavior in a rat model of osteoarthritis pain. Arthritis Res Ther 2022;24:168. 35842727 10.1186/s13075-022-02856-0 PMC9287929

[27] Ivanavicius SP Ball AD Heapy CG Westwood RF Murray F Read SJ. Structural pathology in a rodent model of osteoarthritis is associated with neuropathic pain: increased expression of ATF-3 and pharmacological characterisation. PAIN 2007;128:272–82. 17276007 10.1016/j.pain.2006.12.022

[28] Kelly S Dunham JP Murray F Read S Donaldson LF Lawson SN. Spontaneous firing in C-fibers and increased mechanical sensitivity in A-fibers of knee joint-associated mechanoreceptive primary afferent neurones during MIA-induced osteoarthritis in the rat. Osteoarthritis Cartilage 2012;20:305–13. 22285737 10.1016/j.joca.2012.01.002

[29] Kidd BL. Osteoarthritis and joint pain. PAIN 2006;123:6–9. 16714085 10.1016/j.pain.2006.04.009

[30] Kim YS Anderson M Park K Zheng Q Agarwal A Gong C Saijilafu Young L He S LaVinka PC Zhou F Bergles D Hanani M Guan Y Spray DC Dong X. Coupled activation of primary sensory neurons contributes to chronic pain. Neuron 2016;91:1085–96. 27568517 10.1016/j.neuron.2016.07.044 PMC5017920

[31] Kwok CHT Kohro Y Mousseau M O'Brien MS Matyas JR McDougall JJ Trang T. Role of primary afferents in arthritis induced spinal microglial reactivity. Front Immunol 2021;12:626884. 33897685 10.3389/fimmu.2021.626884 PMC8058457

[32] Malek N Mlost J Kostrzewa M Rajca J Starowicz K. Description of novel molecular factors in lumbar DRGs and spinal cord factors underlying development of neuropathic pain component in the animal model of osteoarthritis. Mol Neurobiol 2024;61:1580–92. 37731080 10.1007/s12035-023-03619-x PMC10896862

[33] Malfait AM Miller RJ. Emerging targets for the management of osteoarthritis pain. Curr Osteoporos Rep 2016;14:260–8. 27730452 10.1007/s11914-016-0326-z PMC5335491

[34] Mapp PI Sagar DR Ashraf S Burston JJ Suri S Chapman V Walsh DA. Differences in structural and pain phenotypes in the sodium monoiodoacetate and meniscal transection models of osteoarthritis. Osteoarthritis Cartilage 2013;21:1336–45. 23973148 10.1016/j.joca.2013.06.031 PMC3790974

[35] McCollum MM Larmore M Ishihara S Ng LCT Kimura LF Guadarrama E Ta MC Vien TN Frost GB Scheidt KA Miller RE DeCaen PG. Targeting the tamoxifen receptor within sodium channels to block osteoarthritic pain. Cell Rep 2022;40:111248. 36001977 10.1016/j.celrep.2022.111248 PMC9523973

[36] McDonnell A Collins S Ali Z Iavarone L Surujbally R Kirby S Butt RP. Efficacy of the Nav1.7 blocker PF-05089771 in a randomised, placebo-controlled, double-blind clinical study in subjects with painful diabetic peripheral neuropathy. PAIN 2018;159:1465–76. 29578944 10.1097/j.pain.0000000000001227

[37] McDougall JJ O'Brien MS. Analgesic potential of voltage gated sodium channel modulators for the management of pain. Curr Opin Pharmacol 2024;75:102433. 38277942 10.1016/j.coph.2024.102433

[38] McQuay HJ Barden J Moore RA. Clinically important changes-what's important and whose change is it anyway? J Pain Symptom Manage 2003;25:395–6. 12727031 10.1016/s0885-3924(03)00099-x

[39] Miller RE Tran PB Ishihara S Syx D Ren D Miller RJ Valdes AM Malfait AM. Microarray analyses of the dorsal root ganglia support a role for innate neuro-immune pathways in persistent pain in experimental osteoarthritis. Osteoarthritis Cartilage 2020;28:581–92. 31982564 10.1016/j.joca.2020.01.008 PMC7214125

[40] Miller RE Tran PB Obeidat AM Raghu P Ishihara S Miller RJ Malfait AM. The role of peripheral nociceptive neurons in the pathophysiology of osteoarthritis pain. Curr Osteoporos Rep 2015;13:318–26. 26233284 10.1007/s11914-015-0280-1 PMC4596062

[41] Mogil JS. Sex differences in pain and pain inhibition: multiple explanations of a controversial phenomenon. Nat Rev Neurosci 2012;13:859–66. 23165262 10.1038/nrn3360

[42] Mousseau M Burma NE Lee KY Leduc-Pessah H Kwok CHT Reid AR O'Brien M Sagalajev B Stratton JA Patrick N Stemkowski PL Biernaskie J Zamponi GW Salo P McDougall JJ Prescott SA Matyas JR Trang T. Microglial pannexin-1 channel activation is a spinal determinant of joint pain. Sci Adv 2018;4:eaas9846. 30101191 10.1126/sciadv.aas9846 PMC6082646

[43] Okun A Liu P Davis P Ren J Remeniuk B Brion T Ossipov MH Xie J Dussor GO King T Porreca F. Afferent drive elicits ongoing pain in a model of advanced osteoarthritis. PAIN 2012;153:924–33. 22387095 10.1016/j.pain.2012.01.022 PMC3313555

[44] Orita S Ishikawa T Miyagi M Ochiai N Inoue G Eguchi Y Kamoda H Arai G Toyone T Aoki Y Kubo T Takahashi K Ohtori S. Pain-related sensory innervation in monoiodoacetate-induced osteoarthritis in rat knees that gradually develops neuronal injury in addition to inflammatory pain. BMC Musculoskelet Disord 2011;12:134. 21679434 10.1186/1471-2474-12-134 PMC3142251

[45] Ovsepian SV Waxman SG. Gene therapy for chronic pain: emerging opportunities in target-rich peripheral nociceptors. Nat Rev Neurosci 2023;24:252–65. 36658346 10.1038/s41583-022-00673-7

[46] Pan B Yu H Fischer GJ Kramer JM Hogan QH. Dorsal root ganglionic field stimulation relieves spontaneous and induced neuropathic pain in rats. J Pain 2016;17:1349–58. 27687223 10.1016/j.jpain.2016.09.004

[47] Pfirrmann CW Oberholzer PA Zanetti M Boos N Trudell DJ Resnick D Hodler J. Selective nerve root blocks for the treatment of sciatica: evaluation of injection site and effectiveness—a study with patients and cadavers. Radiology 2001;221:704–11. 11719666 10.1148/radiol.2213001635

[48] Pomonis JD Boulet JM Gottshall SL Phillips S Sellers R Bunton T Walker K. Development and pharmacological characterization of a rat model of osteoarthritis pain. PAIN 2005;114:339–46. 15777859 10.1016/j.pain.2004.11.008

[49] Power JD Perruccio AV Gandhi R Veillette C Davey JR Syed K Mahomed NN Rampersaud YR. Neuropathic pain in end-stage hip and knee osteoarthritis: differential associations with patient-reported pain at rest and pain on activity. Osteoarthritis Cartilage 2018;26:363–9. 29326061 10.1016/j.joca.2018.01.002

[50] Qu L Caterina MJ. Enhanced excitability and suppression of A-type K(+) currents in joint sensory neurons in a murine model of antigen-induced arthritis. Sci Rep 2016;6:28899. 27363579 10.1038/srep28899 PMC4929491

[51] Rahman W Dickenson AH. Osteoarthritis-dependent changes in antinociceptive action of Nav1.7 and Nav1.8 sodium channel blockers: an in vivo electrophysiological study in the rat. Neuroscience 2015;295:103–16. 25818052 10.1016/j.neuroscience.2015.03.042 PMC4414363

[52] Raoof R Martin Gil C Lafeber F de Visser H Prado J Versteeg S Pascha MN Heinemans ALP Adolfs Y Pasterkamp J Wood JN Mastbergen SC Eijkelkamp N. Dorsal root ganglia macrophages maintain osteoarthritis pain. J Neurosci 2021;41:8249–61. 34400519 10.1523/JNEUROSCI.1787-20.2021 PMC8482866

[53] Schäfers M Lee DH Brors D Yaksh TL Sorkin LS. Increased sensitivity of injured and adjacent uninjured rat primary sensory neurons to exogenous tumor necrosis factor-alpha after spinal nerve ligation. J Neurosci 2003;23:3028–38. 12684490 10.1523/JNEUROSCI.23-07-03028.2003 PMC6742101

[54] Schuelert N McDougall JJ. Involvement of Nav 1.8 sodium ion channels in the transduction of mechanical pain in a rodent model of osteoarthritis. Arthritis Res Ther 2012;14:R5. 22225591 10.1186/ar3553 PMC3392791

[55] Shin SM Cai Y Itson-Zoske B Qiu C Hao X Xiang H Hogan QH Yu H. Enhanced T-type calcium channel 3.2 activity in sensory neurons contributes to neuropathic-like pain of monosodium iodoacetate-induced knee osteoarthritis. Mol Pain 2020;16:1744806920963807. 33054557 10.1177/1744806920963807 PMC7570798

[56] Shin SM Itson-Zoske B Cai Y Qiu C Pan B Stucky CL Hogan QH Yu H. Satellite glial cells in sensory ganglia express functional transient receptor potential ankyrin 1 that is sensitized in neuropathic and inflammatory pain. Mol Pain 2020;16:1744806920925425. 32484015 10.1177/1744806920925425 PMC7268132

[57] Shin SM Itson-Zoske B Fan F Xiao Y Qiu C Cummins TR Hogan QH Yu H. Peripherally targeted analgesia via AAV-mediated sensory neuron-specific inhibition of multiple pronociceptive sodium channels. J Clin Invest 2024;134:e170813. 38722683 10.1172/JCI170813 PMC11213509

[58] Shin SM Lauzadis J Itson-Zoske B Cai Y Fan F Natarajan GK Kwok WM Puopolo M Hogan QH Yu H. Targeting intrinsically disordered regions facilitates discovery of calcium channels 3.2 inhibitory peptides for adeno-associated virus-mediated peripheral analgesia. PAIN 2022;163:2466–84. 35420557 10.1097/j.pain.0000000000002650 PMC9562599

[59] Soga M Izumi T Nanchi I Horita N Yamamoto M Kawasaki S Ogawa K Fujita M Morioka Y. Suppression of joint pain in transient receptor potential vanilloid 4 knockout rats with monoiodoacetate-induced osteoarthritis. Pain Rep 2021;6:e951. 34396019 10.1097/PR9.0000000000000951 PMC8357256

[60] Su J Gao T Shi T Xiang Q Xu X Wiesenfeld-Hallin Z Hökfelt T Svensson CI. Phenotypic changes in dorsal root ganglion and spinal cord in the collagen antibody-induced arthritis mouse model. J Comp Neurol 2015;523:1505–28. 25631752 10.1002/cne.23749

[61] Syx D Tran PB Miller RE Malfait AM. Peripheral mechanisms contributing to osteoarthritis pain. Curr Rheumatol Rep 2018;20:9. 29480410 10.1007/s11926-018-0716-6 PMC6599517

[62] Thakur M Dickenson AH Baron R. Osteoarthritis pain: nociceptive or neuropathic? Nat Rev Rheumatol 2014;10:374–80. 24686507 10.1038/nrrheum.2014.47

[63] Thakur M Rahman W Hobbs C Dickenson AH Bennett DL. Characterisation of a peripheral neuropathic component of the rat monoiodoacetate model of osteoarthritis. PLoS One 2012;7:e33730. 22470467 10.1371/journal.pone.0033730 PMC3312347

[64] Tran PB Miller RE Ishihara S Miller RJ Malfait AM. Spinal microglial activation in a murine surgical model of knee osteoarthritis. Osteoarthritis Cartilage 2017;25:718–26. 27646532 10.1016/j.joca.2016.09.007 PMC5354992

[65] van Lieshout LP Domm JM Rindler TN Frost KL Sorensen DL Medina SJ Booth SA Bridges JP Wootton SK. A novel triple-mutant AAV6 capsid induces rapid and potent transgene expression in the muscle and respiratory tract of mice. Mol Ther Methods Clin Dev 2018;9:323–9. 30038936 10.1016/j.omtm.2018.04.005 PMC6054702

[66] Walsh DA McWilliams DF. Mechanisms, impact and management of pain in rheumatoid arthritis. Nat Rev Rheumatol 2014;10:581–92. 24861185 10.1038/nrrheum.2014.64

[67] Wu Q Henry JL. Changes in abeta non-nociceptive primary sensory neurons in a rat model of osteoarthritis pain. Mol Pain 2010;6:37. 20594346 10.1186/1744-8069-6-37 PMC2908067

[68] Wylde V Beswick A Bruce J Blom A Howells N Gooberman-Hill R. Chronic pain after total knee arthroplasty. EFORT Open Rev 2018;3:461–70. 30237904 10.1302/2058-5241.3.180004 PMC6134884

[69] Yu H Fischer G Ferhatovic L Fan F Light AR Weihrauch D Sapunar D Nakai H Park F Hogan QH. Intraganglionic AAV6 results in efficient and long-term gene transfer to peripheral sensory nervous system in adult rats. PLoS One 2013;8:e61266. 23613824 10.1371/journal.pone.0061266 PMC3628918

[70] Zhuo M Wu G Wu LJ. Neuronal and microglial mechanisms of neuropathic pain. Mol Brain 2011;4:31. 21801430 10.1186/1756-6606-4-31 PMC3163530

[71] Zolio L Lim KY McKenzie JE Yan MK Estee M Hussain SM Cicuttini F Wluka A. Systematic review and meta-analysis of the prevalence of neuropathic-like pain and/or pain sensitization in people with knee and hip osteoarthritis. Osteoarthritis Cartilage 2021;29:1096–116. 33971205 10.1016/j.joca.2021.03.021

