# Supplementary material for: Sensory neuron–specific block of multifaceted sodium channels mitigates neuropathic pain behaviors of osteoarthritis
Source: Pain Rep. 2025 May 27;10(4):e1288. doi: 10.1097/PR9.0000000000001288 (PMC12119052; doi:10.1097/PR9.0000000000001288)
Supplement: SUPPLEMENTARY MATERIAL [file painreports-10-e1288-s001.pdf]

Supplemental materials

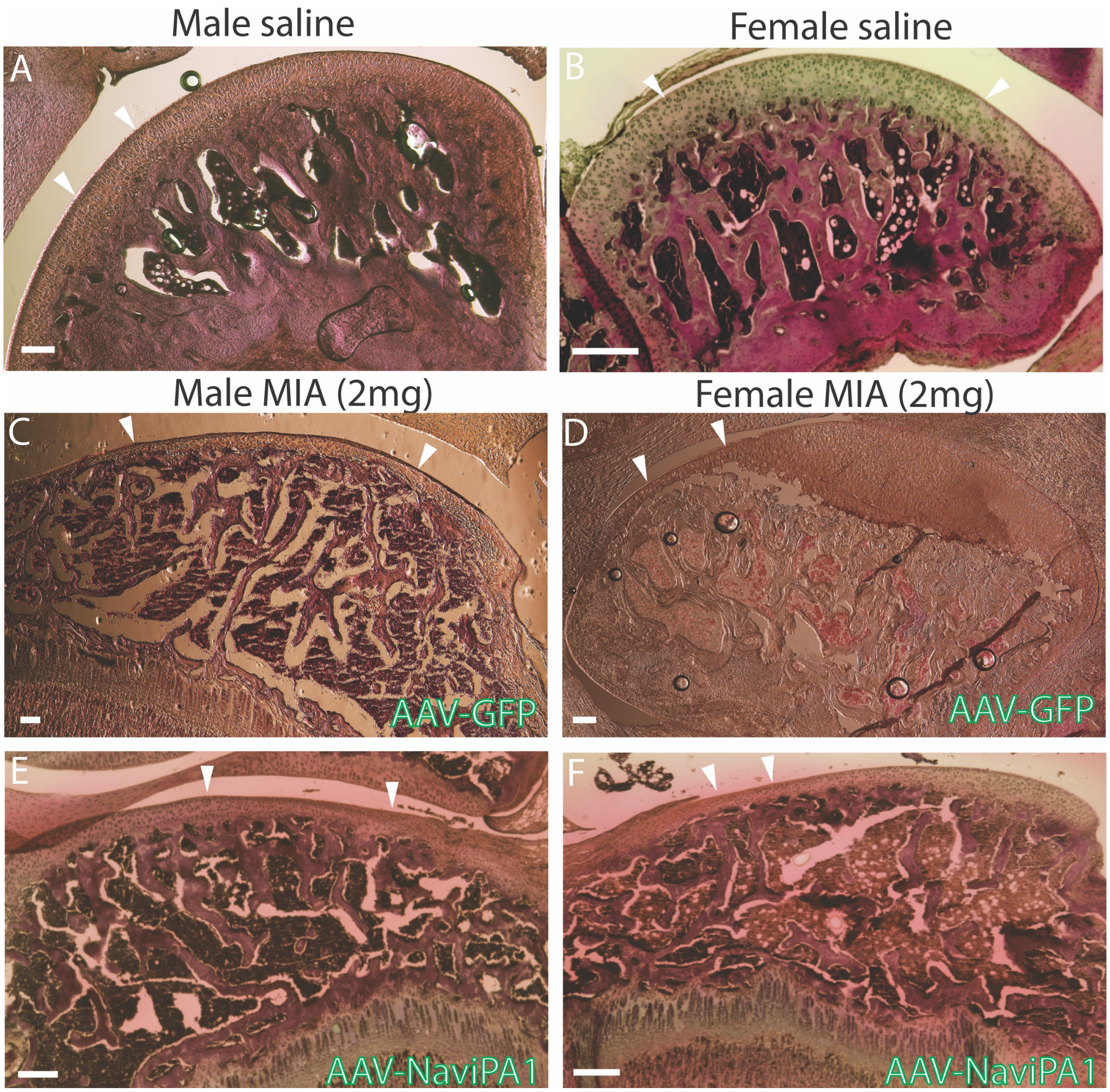

Supple. Fig. 1. **MIA OA knee histopathology.** Representative H&E-stained sagittal section of saline-injected knees, showing full-depth normal cartilage and normal subchondral bone structure in male and female rats (**C and B**). OA-like findings in the representative H&E-stained sagittal sections of the knee 8 weeks after MIA (2mg) and 6 weeks after DRG injection of AAV-GFP (**C** and **D**) and of AAV-GFPNaviPA1 (**E** and **F**), showing comparable articular cartilage loss (arrowheads) and subchondral bone collapse in AAV-GFPNaviPA1 to control AAV-GFP in both male and female rats. Scale bar: 100 μm for all.

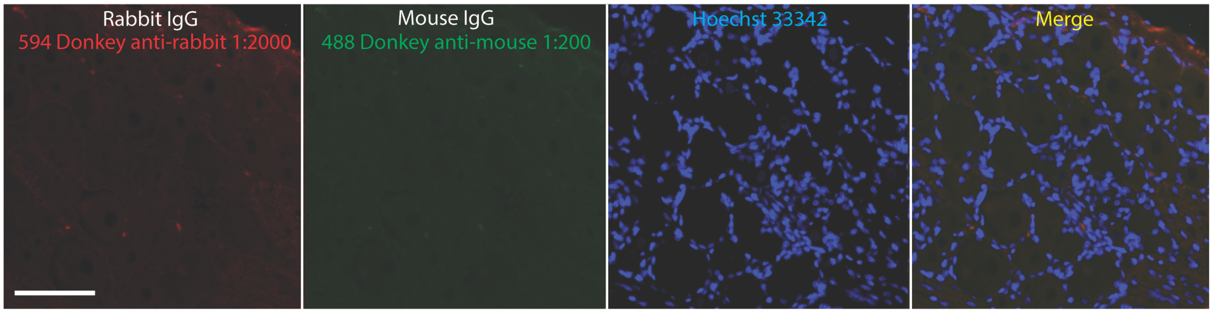

Supple. Fig. 2. IHC staining second antibody (no primary antibody) controls. Normal mouse immunoglobulin G (IgG) (Invitrogen 31903, 1:200) and rabbit IgG (Invitrogen MA5-16384, 1:200) were replaced for the first antibody as the negative controls. The appropriate fluorophore-conjugated (Alexa 488 or Alexa 594, 1:2000) secondary antibodies (Jackson ImmunoResearch, West Grove, PA) were used, respectively. 1.0□g/ml Hoechst33342 (Hoechst, ThermoFisher) was added to the secondary antibody mixture to stain nuclei. Scale bar: 50□m.

Method 2.6.2. IHC and Quantitation

The formalin-fixed, paraffin-embedded tissue sections were deparaffinized, hydrated, and treated by heat-induced antigen epitope retrieval in 10 mM citrate buffer, pH 6.0. Non-specific binding was reduced by incubating the sections for 30 min with a solution of 5% bovine serum albumin (BSA) in PBS plus 0.05% Tween20 (PBST). Samples were first immunolabeled with the selected primary antibodies: mouse GFP (1:500, Santa Cruz Biotechnology, SCB, CA. sc9996), rabbit GFP (1:500, Cell Signaling, Danvers, MA. 2555), rabbit Na$_V$1.7 (1:400, Alomone, Jerusalem, Israel. ASC008), rabbit Na$_V$1.6 (1:400, Alomone, ASC009), rabbit glial fibrillary acidic protein (GFAP, 1:1000, Dako, CA, Z0334), mouse NF200 (1:1000, Sigma-Aldrich, N5389), and mouse b3Tubulin (Tubb3, 1:500, SCB, sc-80016). in a humid atmosphere overnight at 4□C. All antibodies were diluted in PBST, containing 0.05% Triton X-100 and 5% BSA. Normal immunoglobulin G (IgG from the same species as the first antibody was replaced for the first antibody as the negative control. The appropriate fluorophore-conjugated (Alexa 488 or Alexa 594, 1:2000) secondary antibodies (Jackson ImmunoResearch, West Grove, PA) were used to reveal immune complexes. To avoid false-positive results attributable to cross-binding in double-label combinations, each primary antibody raised in a different species was used for double labeling. The immunostaining was examined, and images were captured using a Nikon TE2000-S fluorescence microscope (El Segundo, CA) with filters suitable for selectively detecting the green and red fluorescence using a QuantiFire digital camera (Optronics, Ontario, NY). For double-labeling colocalization, images from the same specimen but showing different antigen signals were overlaid by digitally merging the captured images. NIH ImageJ software (http://rsbweb.nih.gov/ij/) was used for analysis.

IHC quantification: Positive antibody immunostaining was defined as the cells having a fluorescence intensity greater than the average background fluorescence plus 2 standard deviations of the cells in a section of negative control (the first antibody omitted) under identical acquisition parameters (n=10 for different markers), identified by Hoechst counterstain at a different wavelength [83]. For quantification of GFPNa$_V$iPA1 transduction efficiency, every fifth DRG section was selected from the consecutive serial sections (3 to 4 sections for each DRG), and in each selected section, the numbers of GFP labeled cells were counted and transduction efficiency was expressed as the percentage of total neuronal profiles revealed by Tubb3 staining.

Method: 2.7.2. Whole-cell current-clamp recording of dissociated DRG neurons

Whole-cell current-clamp recording of dissociated DRG neurons was performed to determine the effects of AAV-mediated Na_ViPA1 expression on neuronal excitability. Dissociated small- and medium-sized DRG neurons (<35□m in diameter) from saline-injected animals and rats with MIA only, and dissociated DRG neurons with clear GFP expression from MIA rats injected with AAV6.2FF-NP or AAV6.2FF-Na_ViPA1 at 6-week after vector injection were used for recording (n=4~5 rats per group). For whole-cell current-clamp, patch electrodes had a resistance of 0.7–1.5 MΩ when filled with the pipette solution, which contained the following (in mM): 140 K-gluconate, 5 KCl, 2 $MgCl_2$, 0.2 EGTA, 10 HEPES, 4 Mg-ATP, and 0.3 Na^{2+}-GTP, 10 Na2-phosphocreatine pH 7.2 with KOH and osmolarity of 296 to 300 mOsm. The extracellular solution contained the following (in mM): 140 NaCl, 4 KCl, 2 $CaCl_2$, 2 $MgCl_2$, 10 D-glucose, 10 HEPES at pH of 7.4 with NaOH and an osmolarity of 300 mOsm. Whole-cell configuration was obtained in the voltage-clamp mode before proceeding to the current-clamp recording mode. The membrane input resistance was calculated by dividing the end amplitude of steady-state hyperpolarizing voltage deflection by the injected current. Action potentials (APs) were generated by injection of a series of current pulses (100 to 1000 pA in steps of 100 pA, 300 ms). The baseline membrane potential was recorded for 20 ms before the stimulus pulses were injected into the neurons. We defined the resting membrane potential (RMP) as the mean value of the 20 ms pre-stimulus membrane potential in the first trial and the AP rheobase as the minimum depolarizing current required to evoke the first AP. Given the knowledge that nerve injury induces high RMP and low rheobase in DRG neurons, the neurons with stable RMP more negative than -45 mV and overshooting APs (>80 mV RMP to peak) were used for additional data collection. AP frequency, an indicator of PSN excitability, was determined by quantifying the number of APs elicited in response to depolarizing current injections (300 ms).
